# The phosducin-like protein PhLP1 impacts regulation of glycoside hydrolases and light response in *Trichoderma reesei*

**DOI:** 10.1186/1471-2164-12-613

**Published:** 2011-12-19

**Authors:** Doris Tisch, Christian P Kubicek, Monika Schmoll

**Affiliations:** 1 Research Area of Gene Technology and Applied Biochemistry, Institute for Chemical Engineering, Vienna University of Technology, Gumpendorferstraße 1a, A-1060 Wien, Austria

## Abstract

**Background:**

In the biotechnological workhorse *Trichoderma reesei *(*Hypocrea jecorina*) transcription of cellulase genes as well as efficiency of the secreted cellulase mixture are modulated by light. Components of the heterotrimeric G-protein pathway interact with light-dependent signals, rendering this pathway a key regulator of cellulase gene expression.

**Results:**

As regulators of heterotrimeric G-protein signaling, class I phosducin-like proteins, are assumed to act as co-chaperones for G-protein beta-gamma folding and exert their function in response to light in higher eukaryotes. Our results revealed light responsive transcription of the *T. reesei *class I phosducin-like protein gene *phlp1 *and indicate a light dependent function of PhLP1 also in fungi. We showed the functions of PhLP1, GNB1 and GNG1 in the same pathway, with one major output being the regulation of transcription of glycoside hydrolase genes including cellulase genes in *T. reesei*. We found no direct correlation between the growth rate and global regulation of glycoside hydrolases, which suggests that regulation of growth does not occur only at the level of substrate degradation efficiency.

Additionally, PhLP1, GNB1 and GNG1 are all important for proper regulation of light responsiveness during long term exposure. In their absence, the amount of light regulated genes increased from 2.7% in wild type to 14% in Δ*phlp1*. Besides from the regulation of degradative enzymes, PhLP1 was also found to impact on the transcription of genes involved in sexual development, which was in accordance with decreased efficiency of fruiting body formation in Δ*phlp1*. The lack of GNB1 drastically diminished ascospore discharge in *T. reesei*.

**Conclusions:**

The heterotrimeric G-protein pathway is crucial for the interconnection of nutrient signaling and light response of *T. reesei*, with the class I phosducin-like protein PhLP1, GNB1 and GNG1 acting as important nodes, which influence light responsiveness, glycoside hydrolase gene transcription and sexual development.

## Background

Fungi are exposed to different external abiotic factors during their life cycle. Like almost every organism, fungi can adapt to the abiotic factor light by considerable adjustments of their physiology, including metabolic processes [1-3]. Light has an effect on almost all metabolic pathways in fungi, such as carotenoid metabolism [4], fatty acid metabolism [5], cAMP levels [6], sulfur metabolism [7] and carbohydrate metabolism [8]. It was reported already more than 40 years ago that consuming available nutrients and sensing of light are connected - it depends on the carbon source if light exerts a stimulating effect on growth rates (reviewed in [9]). The number of genes regulated in response to light ranges from only one in *Cryptococcus neoformans *[10] to several hundred in *Neurospora crassa *(314 genes; [11,12]) and *Trichoderma atroviride *(2.8% of genes; [13]) up to 5% of the whole genome in *Aspergillus nidulans *[14].

Due to the immobility of a fungus, the signal transduction system is important for survival under changing environmental conditions [15]. Being crucial for successful competition in nature, transmission of nutrient signals can be considered responsible for integration with light signaling, resulting in adjustment of the growth rate in light and darkness to environmental conditions. One prominent mechanism for achievement of this task is the heterotrimeric G-protein signaling pathway [16,17], which is interconnected with the light response pathway in *Trichoderma reesei *[18-21].

*Trichoderma reesei *(*Hypocrea jecorina*) was discovered in the 1940s and this fungus is most prominent for its ability to produce cellulases. These enzymes are used in various biotechnological processes, such as in the textile industry, food and feed industry [22-26] and heterologous protein production [27]. Despite its long history of use for research and industry, sexual development under laboratory conditions was only recently achieved [28] and only a few details on the mechanism and regulation of mating in *T. reesei *are available so far [29]. Concerns about the consumption of fossil fuels, the CO_2 _balance and imminent climate change prompted increased research efforts towards second generation biofuels and hence towards improved cellulase production. The sequence analysis of the genome of *T. reesei *reveals that the genome comprises 16 hemicellulase genes and nine cellulase genes - surprisingly low numbers for the currently most important cellulase producer worldwide [30]. Induction and regulation of the genes responsible for this industrial application have been studied for decades [31,32]. However, the impact of signal transduction pathways on biosynthesis of degradative enzymes and their responsiveness to light only recently received attention [21].

Albeit unexpected for an important biotechnological workhorse, several *Trichoderma *species were used as photomorphogenic models [33] and since the discovery of light modulated cellulase gene expression [34] the interrelationship between metabolic functions and light response became a focus of research. Like many other metabolic processes, the production of cellulases is influenced by light and the light regulatory protein ENVOY as well as the two photoreceptors BLR1 and BLR2 (Blue Light Receptors 1 and 2) in *T. reesei *[21,34,35]. Investigation of the transmission of environmental signals by the G-protein alpha subunits revealed an effect of this signaling pathway on cellulase gene expression. The signals transmitted by both GNA1 and GNA3 have a positive impact on cellulase gene expression, which is dependent on light [18,20,21]. The fact that these G alpha subunits are involved in light modulated cellulase gene transcription raises the question, as to how the light signal could be transferred to and/or integrated with the nutrient signal transmitted via the heterotrimeric G-protein pathway. In *T. reesei *several regulatory mechanisms impacting heterotrimeric G-protein signaling are present [36].

A light-dependent function for phosducin-like proteins has previously been shown in higher eukaryotes: Phosducins (PHDs) were first isolated in photoreceptor cells of the retina of mammals [37,38], where they act as regulators of G-protein signaling [39-41]. Among the three classes of phosducins and phosducin-like proteins (PhLPs), phosducins of class I play a role in the G beta-gamma binding [42] by acting as co-chaperones in their folding and are required for efficient G-protein signaling [39,40,43,44]. Knock-out mutants of class II phosducins in *Saccharomyces cerevisiae *were not able to survive, suggesting that class II phosducins are essential for cell growth [45]. PHDs and PhLPs of class III are assumed to play a role in actin folding [46]. In fungi the function of phosducin-like proteins in G-protein beta-gamma assembly was confirmed [47-49]. Disruption of the *Cryphonectria parasitica *class I phosducin BDM-1 impacts on the accumulation of the G alpha subunit CPG-1 [47] and is a probable casein kinase 2 target [49]. The *Aspergillus nidulans *phosducin homologue PhnA was shown to be involved in regulation of the sterigmatocystin biosynthesis pathway [48]. Nevertheless, a related function in light response in fungi has not been explored so far. The genome of *T. reesei *comprises two genes encoding phosducin-like proteins of classes I and II, both of which have orthologues in several other fungi, including *Aspergilli *and *Neurospora *[36]. Besides the phosducin-like proteins, also microbial opsins as G-protein coupled receptors can be considered promising candidates for connecting components between nutrient and light signaling [50,51]. However, in the *T. reesei *genome no microbial opsins were detected [36].

Our study revealed that class I phosducin-like proteins are involved in transmission of light-dependent signals in fungi. Transcription of *phlp1 *was responsive to light and PhLP1 acted in the same pathway as the G-protein beta and gamma subunits GNB1 and GNG1. Genome wide transcriptional analysis showed considerable light dependent gene regulation, especially of glycoside hydrolase genes, which was at least in part mediated by PhLP1. We identified a broad positive influence of PhLP1-GNB1-GNG1 on gene expression in light, suggesting sustainment of predominantly nutritional processes by this signaling pathway in light. Moreover, we found that PhLP1 positively regulated transcript levels of the peptide pheromone precursor *hpp1 *as well as of the homologue of the yeast pheromone transporter gene *ste6 *and consequently impacted on mating efficiency.

## Results

### Genome-wide analysis of light-dependent transcription in *T. reesei*

To gain an overview on light-dependent physiological processes in *T. reesei*, genome wide transcriptional analysis using microarrays was applied (Gene Expression Omnibus accession number GSE27581). *T. reesei *was grown on microcrystalline cellulose for 72 hours in constant light and constant darkness, since the fungus is in its active phase of growth and enzyme production at this time. Constant conditions were chosen in order to avoid interference of circadian rhythmicity with our results. As internal controls we checked genes with known transcriptional patterns under these conditions [29,34,52]. Transcription of the light regulatory protein encoding *env1*, the photolyase gene *phr1 *and the peptide pheromone precursor encoding gene *hpp1 *correlated with earlier data under similar conditions and hence confirm that growth conditions were appropriate for our analysis. Additionally we tested whether the microarray results reflected actual expression patterns in the sample by qRT-PCR of the two major cellulase genes *cel7a/cbh1 *and *cel6a/cbh2*. While transcript abundance of *cel7a/cbh1 *exceeded the saturation threshold (around 65000) of the arrays and could thus not be used as an internal control for appropriate cultivation conditions, transcriptional patterns of the cellulase gene *cel6a/cbh2*, correlated with qRT-PCR data. For both *cel7a/cbh1 *and *cel6a/cbh2 *clear correlation with earlier data [34] was observed (*cel7a/cbh1 *transcript levels in QM9414: +102% +/- 39%; *cel6a/cbh2 *transcript levels in QM9414: +98% +- 13% in light compared to darkness).

**Figure 1:**
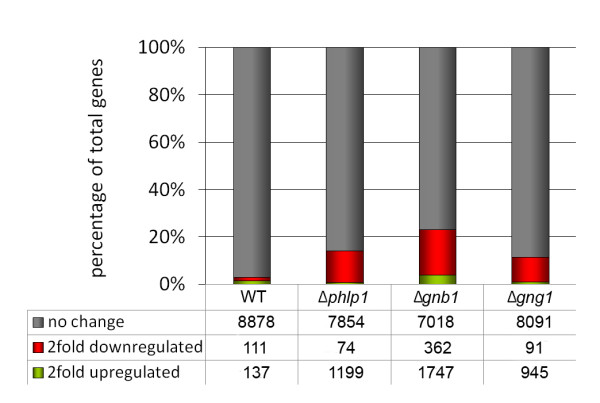
**Impact of light on global gene regulation upon deletion of *phlp1, gnb1 *or *gng1***. Transcript abundances of genes in the respective wild type strain QM9414 (WT) or deletion strains grown in light were compared to transcription in darkness. The figures show the amounts of genes at least two-fold upregulated in light, downregulated in light as compared to darkness or unchanged upon growth in light or darkness (i. e. below the threshold of two-fold). Number of total genes of the respective characteristics is given below the diagram in the table for every condition and strain.

*T. reesei *showed considerable alterations in gene regulation in dependence on the light status (Figure 1; Additional file 1, Dataset 1). 248 genes (2.7% of total genes) were found to be at least two-fold differentially regulated under these conditions, which is in the range of light regulated genes found in other ascomycetes [11-14]. Gene set enrichment analysis (GSEA; [53]) was used to elucidate which processes were specifically enhanced or decreased in light (Additional file 2, Table S1A and S1B). As expected, genes representing functions in DNA photolyase activity were significantly enriched among the genes upregulated in light. Surprisingly, we found significant enrichment (p-value ≤ 0.005) of genes involved in carbohydrate metabolic processes (enrichment score (ES) = 25.03), cellulase activity and cellulose binding (ES = 11.72 and 36.0 respectively), regulation of oxidoreductase activity (ES = 25.41) and sulphate transport (ES = 11.72) in this group. These results were in agreement with earlier data on light-modulated cellulase gene expression [34,35] and an interconnection with sulphate metabolism and light response in *T. reesei *[7]. In contrast, genes involved in lipid biosynthetic processes (ES = 14.66) and transport processes (ES = 9.99) were downregulated in light.

Specifically, among the group of 137 genes, which were at least two-fold upregulated in constant light, we found eight genes involved in sexual development (*ste2*, TR_109078, TR_124222, TR_31134, TR_62693 = *ste6, mata1*, TR_59364, *hpp1*), hence supporting the requirement of light for this process [28]. Considering the biotechnological significance of *T. reesei *in production of cellulolytic enzymes, detection of 16 glycoside hydrolase (GH) genes, which were upregulated in light, supports the hypothesis of a high importance of light in regulation of extracellular substrate degradation (Additional file 1, Dataset 1). Those GH-genes included the two major cellulase genes *cel7a/cbh1 *and *cel6a/cbh2*, the endoglucanase *cel5a/egl2*, the xylanase *xyn2 *and the beta-xylosidase *bxl1*. Interestingly, we also found transcription of *cel61a/egl4 *and an additional GH-family 16 gene to be decreased in constant light, suggesting that *T. reesei *adjusts its cellulase mixture to different physiological requirements in light and darkness.

### The class I phosducin-like protein is responsive to light

The widespread influence of light on glycoside hydrolase gene expression suggested a close interconnection between light response and nutrient signaling, which was also corroborated by our studies on G-protein signaling [18,20,21]. As regulators of G-protein signaling, class I phosducin-like proteins, which are involved in transmission of light signals in higher eukaryotes [44], represent viable candidates as nodes between the pathways transmitting light and nutrient signals.

**Figure 2:**
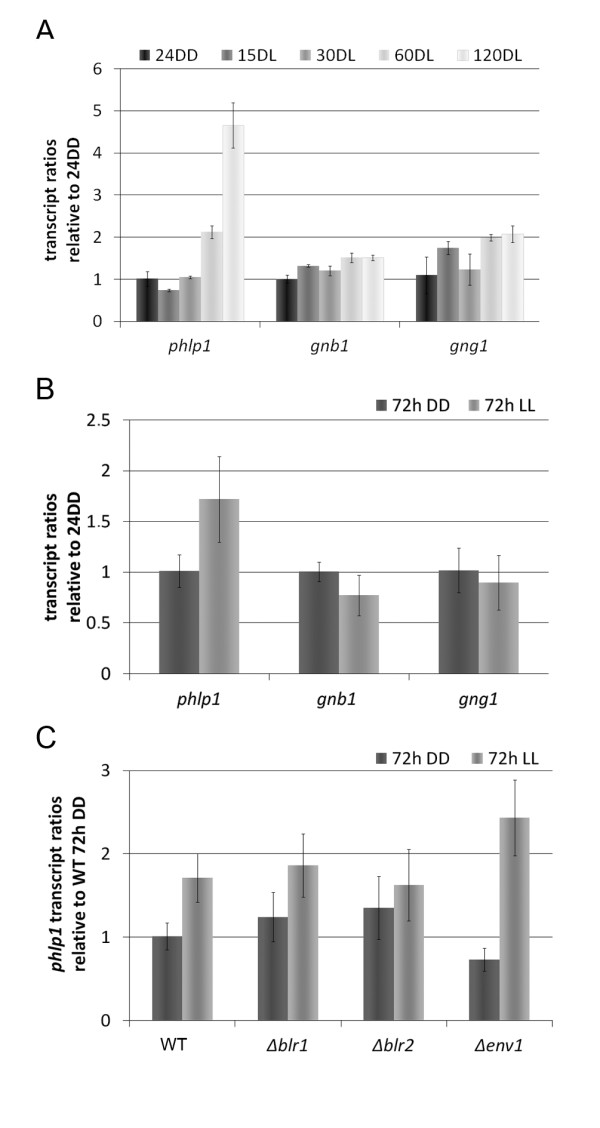
**Light response of *phlp1, gnb1 *and *gng1 *transcripts**. Transcript levels of *phlp1, gnb1 *and *gng1 *were analyzed by qPCR. A: After cultivation of the wild type strain QM9414 (WT) on Mandels-Andreotti minimal media with 1% (w/v) of glycerol as carbon source for 24 hours in darkness (24 DD) mycelia were exposed to light for 15 minutes (15 DL), 30 minutes (30 DL), 60 minutes (60 DL) or 120 minutes (120 DL), respectively. B: WT was cultivated on Mandels-Andreotti minimal media with 1% (w/v) of cellulose as carbon source for 72 hours in constant darkness (DD) or constant light (LL). C: WT, the deletion strains of the blue light regulators 1 and 2 (Δ*blr1 *and Δ*blr2) *and the light regulatory protein ENVOY (Δ*env1*) were cultivated under the same conditions as described in B and *phlp1 *transcript levels were measured.

We therefore first tested short-term light responsiveness of the class I phosducin-like protein encoding gene *phlp1 *(Phosducin-like Protein 1; [GenBank: EGR50146.1]). *phlp1 *was significantly induced by light after 60 minutes of exposure (Figure 2A) and also upregulated in light upon growth on cellulose and long term light exposure (Figure 2B). In *N. crassa *[12] and *A. nidulans *[14] orthologues of *phlp1 *were not found among light regulated genes. Following the definition by Chen and co-workers [12], *phlp1 *belongs to the group of late light responsive genes (LLRGs). This classification hints to a function of *phlp1 *also upon prolonged cultivation in light, i. e. under constant conditions of growth on cellulose in light or darkness. Indeed, transcript abundance of *phlp1 *is increased in light after 72 h of growth compared to darkness (Figure 2B).

The sequence of a light response element (LRE 5'-GATcC-N_50_- CGATc 3'; [54]), which is a probable binding site for the WCC complex in *N. crassa*, was identified 485 bp upstream of the translational start codon. The core region of an ELRE (Early Light Response Element) defined by Chen [12] superposes this element. This fact supports a light responsive role for *phlp1 *in *T. reesei*. However, the light responsive element (ATCG) described by [55] does not entirely correspond to the motif identified by He [54] and was not detected in the 1 kb promotor region of *phlp1*. Accordingly, the *phlp1 *orthologue of *N. crassa *was not found to be a target of the white collar complex [55].

Class I phosducin like proteins are supposed to act as co-chaperones for G-protein beta and gamma subunit folding [44]. In contrast to *phlp1*, transcript abundance of the genes encoding the G-protein beta and gamma subunits (*gnb1 *and *gng1*; [36]) showed only a small short term response to illumination on glycerol and no change under constant long-term conditions in light compared to darkness whilst grown on cellulose (Figure 2A,B). To gain insight into the mechanism of light regulation of *phlp1*, we analyzed transcript ratios of *phlp1 *in the photoreceptor mutants Δ*blr1*, Δ*blr2 *and Δ*env1 *(Figure 2C) under constant conditions of growth on cellulose. Deletion of *env1 *caused a significant increase in light responsiveness of *phlp1 *transcription (transcript abundance in light compared to darkness) compared to the parental strain QM9414 (Figure 2C). In contrast, lack of BLR1 or BLR2 resulted in only minor differences of transcript abundance compared to wild type, which does not corroborate binding of a photoreceptor complex to the LRE motif in the *phlp1 *promotor. However, in the respective mutants the detected differences between light and darkness lack statistical significance (Figure 2C), which may be interpreted as abolished light response. Hence, although a certain influence of the photoreceptors BLR1 and BLR2 on transcription of *phlp1 *was observed, the function of ENV1 in regulation of *phlp1 *is likely more relevant with respect to light response.

### Deletion mutants of *phlp1, gnb1 *and *gng1 *display similar phenotypes

**Figure 3:**
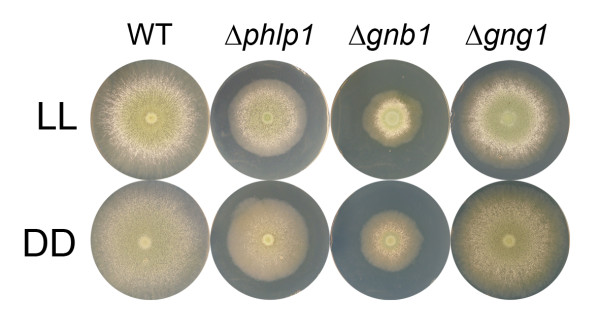
**Relevance of PhLP1, GNB1 or GNG1 for growth on solid media**. Phenotypes of the deletion strains Δ*phlp1*, Δ*gnb1 *and Δ*gng1 *and the parental strain QM9414 (WT) upon growth on malt extract agar plates (3% w/v) in constant light (LL) or darkness (DD) after three days of cultivation at 28°C.

**Figure 4:**
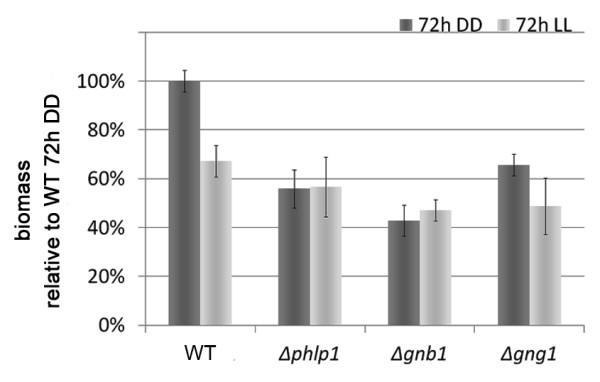
**Biomass formation on cellulose**. Biomass formation upon cultivation on Mandels-Andreotti minimal media with 1% (w/v) microcrystalline cellulose for 72 hours in constant darkness (DD) or constant light (LL) in the wild type QM9414 (WT) and deletion strains.

To confirm the hypothesis that *phlp1 *is involved in the G-protein signaling pathway by acting on the G beta and the G gamma subunits, we constructed deletion mutants of *phlp1, gnb1 *[GenBank: EGR50145.1] and *gng1 *[GenBank: EGR50886.1]. Phenotypes of Δ*phlp1*, Δ*gnb1 *and Δ*gng1 *in constant darkness (DD) and constant light (LL) revealed largely similar growth patterns of the three deletion mutant strains, which clearly differed from the parental strain QM9414 (Figure 3). Evaluation of the number of spores revealed increased sporulation in light in Δ*phlp1*, Δ*gnb1 *and Δ*gng1 *strains and a minor decrease in Δ*phlp1 *in darkness (Additional file 3, Figure S1). Lack of *phlp1, gnb1 *or *gng1 *caused decreased hyphal extension rates and biomass formation under all conditions tested including cellulose (Figure 4; Additional file 3, Figure S2 and S3).

These data suggest a carbon source and light-independent decrease of biomass formation and hence a light-independent growth defect upon lack of PhLP1, GNB1 or GNG1. The strikingly similar phenotypes of strains lacking PhLP1, GNB1 and GNG1 support the expected function of PhLP1 in efficient folding of GNB1 and the hypothesis that PhLP1, GNB1 and GNG1 act in the same pathway.

### Lack of PhLP1, GNB1 or GNG1 causes considerable loss of regulatory control in light

To determine if PhLP1 and its regulatory targets GNB1 and GNG1 would function as a node between the light response pathway and nutrient signaling via the heterotrimeric G-protein cascade, we applied genome wide transcriptional analysis of Δ*phlp1*, Δ*gnb1 *and Δ*gng1 *in light and darkness. We chose cultivation under constant conditions (light or darkness) with cellulose as carbon source, as expression of plant cell wall degrading enzymes mainly produced on cellulose represents the best studied output pathway in *T. reesei*. The function of PhLP1, GNB1 and GNG1 could be on the one hand regulation of light responsiveness (short or long term) of gene expression, which we describe as the difference between transcript abundance in light compared to darkness in the same strain (mutant or wild type). On the other hand, alteration of gene expression in the mutant strains compared to the parental strain is important for determination of the (likely indirect) regulatory targets of the function of PhLP1, GNB1 and GNG1.

Because of the presumed function of PhLP1 in light response, we first investigated the influence of PhLP1, GNB1 and GNG1 on light responsiveness of gene expression. Analysis of transcript profiles in Δ*phlp1*, Δ*gnb1 *and Δ*gng1 *indicated that these genes are crucial for regulation of light responsiveness on gene expression. In these strains not only the roughly 3% of genes were regulated in response to light as in the parental strain QM9414, but this number considerably increased up to 23% of all genes in Δ*gnb1 *(Figure 1). Interestingly, the most striking difference was the high number of genes downregulated in light compared to darkness, which suggests that several processes maintained or regulated by PhLP1, GNB1 and GNG1 in the parental strain in light might not be initiated or enhanced further in their absence, because the light signal does not reach the target. We conclude that the signal transduction components and their output pathways, which trigger regulation of physiological processes, are different in light than in darkness. As candidates for the respective targets we identified glycoside hydrolases, of which up to 51 (in Δ*gnb1*) were downregulated in contrast to two in the parental strain in light, and also components of sulphur metabolism with up to 19 genes downregulated in this strain in contrast to none in the QM9414 strain (Additional file 1, Dataset 1; Additional file 4, Table S2).

### Light regulation of glycoside hydrolases is prevalent in *T. reesei*

In Δ*phlp1*, Δ*gnb1 *and Δ*gng1 *the number of glycoside hydrolases genes differentially regulated in light and darkness was even higher than in the parental strain. While GH encoding genes upregulated in QM9414 are in no case regulated contrarily in the mutants (i. e. downregulated), many GH-encoding genes show lower transcript levels in Δ*phlp1*, Δ*gnb1 *and Δ*gng1 *than in QM9414 (Additional file 1, Dataset 1; Additional file 4, Table S2), which indicates that PhLP1, GNB1 and GNG1 are involved in triggering their expression specifically in light. Intriguingly, only in 10 out of 49 GH-families represented in the *T. reesei *genome, no member was found to be regulated light-dependently in any of the strains tested, among which only the functionalities of rhamnogalacturonyl hydrolase (GH family 105), beta-galactosidase (*bga1*, GH family 35) and a-N-acetylglucosaminidase (GH family 89) are not available within any other GH family.

In total we found 99 GH-encoding genes out of 190 in the genome to be regulated by light in the parental strain QM9414 and/or one or more mutant strains. Hence these genes can be expected to be potentially regulated in response to light and to a nutrient signal as transmitted via the heterotrimeric G-protein pathway. These results are in accordance with earlier studies revealing that the influence of light on growth of a fungus is dependent on the provided carbon source [9,52,56]. Tight regulation of the enzymes required for carbon source utilization in response to light is expected to be required for this mechanism, which is likely to involve the function of PhLP1, GNB1 and GNG1.

### Regulatory targets of PhLP1

**Figure 5:**
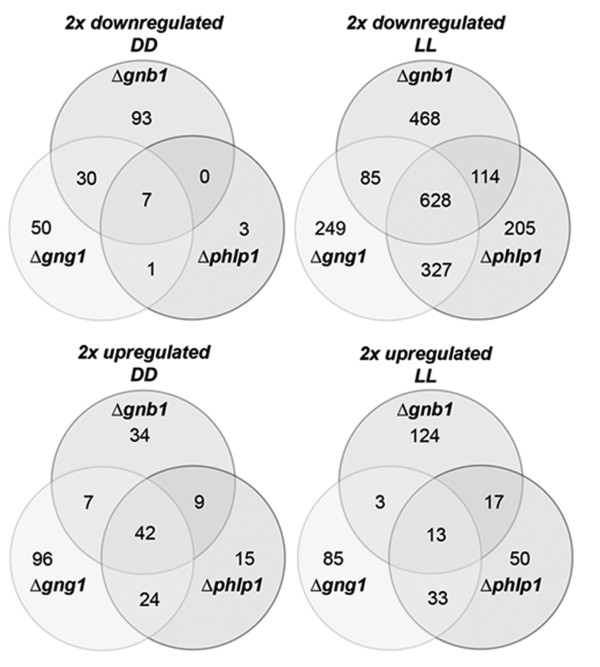
**Regulatory targets of PhLP1, GNB1 and GNG1 in light and darkness**. VENN diagram of genes 2fold downregulated genes in the deletion strains Δ*phlp1*, Δ*gnb1 *and Δ*gng1 *in comparison with the parental strain QM9414 (WT) in light and darkness (top) and two-fold upregulated genes in the deletion strains (bottom). For details on gene regulation see Additional files 5, 6 and 7.

As lack of PhLP1, GNB1 and GNG1 revealed considerable light dependent regulation by these factors, we were interested, whether their function is a positive or negative one and if illumination would be relevant for their impact. To this end we compared transcript abundance of genes in mutants with their parental strain upon growth under constant conditions as described above. This analysis allowed us to identify targets of PhLP1, GNB1 and GNG1 (as reflected by differential regulation compared to the parental strain), which might directly or indirectly be involved in light-adaptive processes and regulation of output pathways such as glycoside hydrolase gene expression in *T. reesei *(Additional files 5, 6 and 7). Investigation of the targets of PhLP1 revealed 128 genes to be upregulated at least two-fold in Δ*phlp1 *in light compared to the parental strain (Figure 5; Additional file 5, Dataset 2). Among these were *hfb1 *(TR_73173) and *hfb2 *(TR_119989) - two genes encoding class II hydrophobins and six glycoside hydrolase family proteins. However, the number of genes positively influenced by PhLP1 in light was considerably higher. 1298 genes were downregulated at least two-fold in the *phlp1 *deletion strain (Additional file 5, Dataset 2). In addition to 45 glycoside hydrolase family genes, we found 10 G-protein coupled receptor proteins in this group (TR_5647, TR_81383, TR_45573, TR_62462, TR_41260, TR_111861, TR_57101, TR_72672, TR_55561, TR_59778), of which 5 belonged to the PTH11-like GPCRs reported to play a role in pathogenesis of *Magnaporthe grisea *[57].

**Figure 6:**
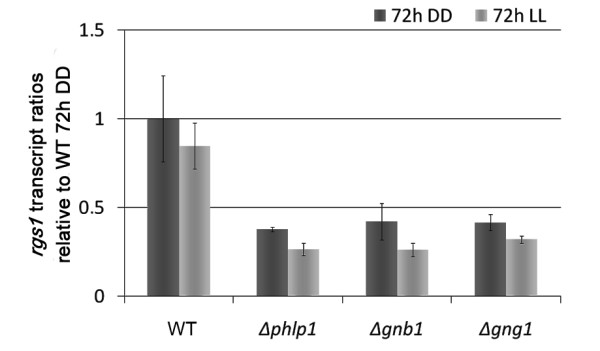
**Transcription of *rgs1***. Transcript ratios of *rgs1 *in QM9414 (WT), Δ*phlp1*, Δ*gnb1 *and Δ*gng1 *relative to WT DD in light and darkness obtained from microarray data. Data were confirmed with qRT-PCR.

In darkness, transcript abundance of 105 genes was enhanced in the Δ*phlp1 *mutant including two glycoside hydrolase family 92 genes, which encode mannosidases and five other candidates for glycoside hydrolases (Additional file 5, Dataset 2). Moreover, a class II hydrophobin gene *hfb4 *(TR_106538, [58]) as well as *ooc1*, which is related to cellulase transcription [59] were found to be targets of PhLP1. Only 10 genes were downregulated in Δ*phlp1 *in darkness and most of them overlap with those downregulated in light (Additional file 5, Dataset 2). 6 genes were significantly downregulated in both light and darkness in the Δ*phlp1 *strain compared to the parental strain QM9414 and hence represent light-independent targets of PhLP1 (Additional file 5, Dataset 2). Interestingly these targets included *rgs1 *(TR_54395, Figure 6 [Genbank: EGR52150.1), a G-protein signaling regulator encoding gene and a gene encoding a GprK-type GPCR (TR_81383), which also comprises an RGS domain.

### Overlapping targets of PhLP1, GNB1 and GNG1

For evaluation of the hypothesis that GNB1, GNG1 and PhLP1 are acting in the same pathway and to elucidate the regulatory output of the respective interactions, we analyzed the target genes common to these components (Figure 5; Additional file 8, Dataset 5). Such genes would reflect a function of all three proteins - presumably due to their complex formation or a tight regulation of each other - in regulation of the respective target genes.

In light, we detected a 10.2% (13 genes) overlap of all upregulated genes and 30.6% (628 genes) overlap of all downregulated genes in Δ*phlp1*, Δ*gnb1 *and Δ*gng1 *(Figure 5). In darkness 42 genes were upregulated in all three mutants, including two glycoside hydrolases (TR_124175 and TR_55886), one putative PTH11-like GPCR (TR_121990), a hydrophobin gene *hfb4 *(TR_106538), a gene involved in conidiation (*cmp1*) and one polyketide synthase (group 4, TR_82208 [60]). Seven genes - among them are *rgs1*, a gene encoding a Regulator of G-protein signaling, and a putative G-protein coupled receptor comprising an RGS-domain (TR_81383) - were downregulated in darkness.

Gene regulation in the individual mutant strains was more similar between Δ*phlp1 *and Δ*gng1 *than between Δ*phlp1 *and Δ*gnb1 *for both up- and downregulated genes in light (Figure 5). Most interestingly, among the 628 genes downregulated in light in all mutant strains, again *rgs1 *was found to be downregulated (Figure 6). Since the positive effect of PhLP1-GNB1-GNG1 on 628 genes in light was the most important effect, we conclude that this group comprises the most crucial target genes of G-protein beta-gamma signaling.

As expected based on the results described above, the predominant functional group within 628 positive targets in light were the glycoside hydrolases with 21 target genes (Additional file 8, Dataset 5). Additionally, 2 PTH11-type GPCR encoding genes (TR_57101 and TR_5647), and one GprK-type GPCR containing an RGS-domain (TR_81383), 2 hydrophobin genes *hfb5 *and *hfb6 *(TR_104293 and TR_105869, [58]), a polyketide synthase group 7 encoding gene (TR_65116, [60]) and two non-ribosomal peptide synthase genes (TR_24586 and TR_71005), two putative multicopper oxidase genes (TR_54239 and TR_102820) as well as six putative transcription factors (TR_26163, TR_104380, TR_106654, TR_107974, TR_110901 and TR_111145) were found to be targets of PhLP1-GNB1-GNG1. Interestingly, downregulation of four genes (TR_119857, TR_122371, TR_81383 and *rgs1*) was found to be independent of the light status.

We conclude that the PhLP1-GNB1-GNG1 complex impacts signal perception by G-protein coupled receptors, transmission and termination of the signals by regulation of G-protein alpha subunit activity, transcriptional activity of target genes and hence the output in terms of carbohydrate utilization and secondary metabolism.

### PhLP1, GNB1 and GNG1 regulate cellulase gene expression

**Figure 7:**
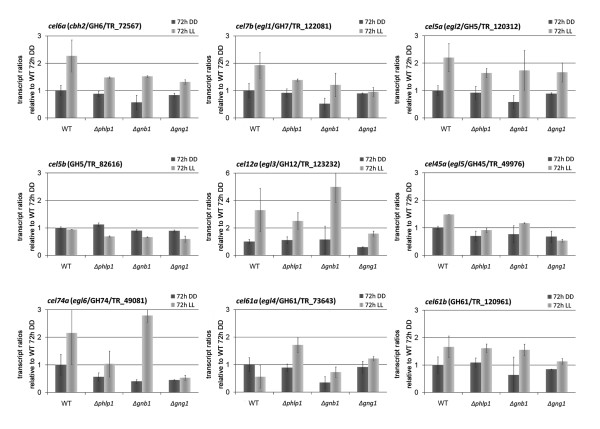
**Transcript levels of genes encoding endo- and exoglucanases**. Transcript profiles of all endo- and exoglucanase genes of *T. reesei *[30] are shown except for *cel7a*/*cbh1*, which exceeded the saturation limit in microarray experiments. Data are shown relative to QM9414 (WT) DD levels. Transcript levels are given as means of two independent biological replicates with standard deviations.

**Figure 8:**
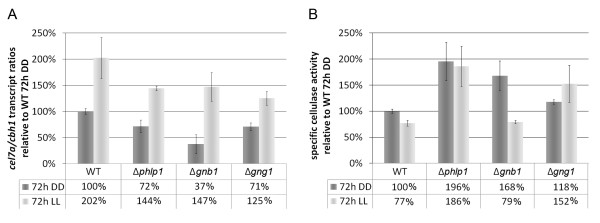
**Relevance of PhLP1, GNB1 and GNG1 for cellulase regulation**. The deletion strains Δ*phlp1*, Δ*gnb1 *and Δ*gng1 *and the parental strain QM9414 were cultivated on Mandels-Andreotti minimal media with 1% (w/v) microcrystalline cellulose for 72 hours in constant darkness (DD) or constant light (LL). A: *cel7a/cbh1 *transcript levels were determined by qPCR and related to the wild type QM9414 (WT) in darkness (DD). The table below the diagram shows the percentage of transcript abundance related to WT grown for 72 hours in darkness. B: FPU related to biomass is given as relative values compared to WT in darkness (DD). The table below the diagram shows the percentage of specific cellulase activity related to WT grown for 72 hours in darkness.

Since our genome wide analysis revealed a crucial function of PhLP1, GNB1 and GNG1 in regulation of hydrolytic enzymes, we investigated the subset of these genes responsible for degradation of cellulosic substrates in more detail. In addition to *cel7a/cbh1*, for seven out of nine cellulases (*cel6a/cbh2 *(TR_72567), *cel7b/egl1*, (TR_122081), *cel5a/egl2*, (TR_120312), *cel12a/egl3*, (TR_123232), *cel45a/egl5*, (TR_49976), cel74a/*egl6*, (TR_49081), *cel61b *(TR_120961)), including a gene encoding a cellulase enhancing protein of GH family 61, we observed a positive effect of light on transcript levels in at least one of the strains tested, which is reduced in the Δ*phlp1 *and Δ*gng1 *deletion strains, as was observed for *cel7a/cbh1 *(Figure 7 and 8). A negative effect of light was not detected for any cellulase gene. Moreover, in total eleven out of 16 hemicellulase genes [30] are differentially regulated at least two-fold in light and darkness in QM9414 and/or the mutant strains (Additional file 1, Dataset 1; Additional file 4, Table S2).

qRT-PCR analysis of transcription of *cel7a/cbh1 *showed consistently decreased levels in Δ*phlp1*, Δ*gnb1 *and Δ*gng1 *in light compared to the parental strain QM9414 (by 28.7% +- 2.3% for Δ*phlp1*, by 27.4% +- 13.4% for Δ*gnb1 *and by 38.1% +- 6.4% for Δ*gng1*) (Figure 8A). Transcription abundance in the deletion strains was not only observed in light, but also in darkness. In particular, in the Δ*phlp1 *strain transcript levels were reduced by 28.5% +- 12.0%, in the Δ*gng1 *strain by 29.5% +- 7.7% and in the Δ*gnb1 *strain even by 62.8% +- 18.2% compared to QM9414 in darkness. Considering the positive effect of GNA1 and GNA3 on cellulase expression [18,20], this result is in accordance with a positive effect of phosducin-like proteins on the efficiency of G-protein signaling [44]. Analysis of specific cellulase activity in Δ*phlp1*, Δ*gnb1 *and Δ*gng1 *revealed increased efficiency of the cellulase mixtures secreted by these strains as compared to the parental strain (Figure 8B), hence reflecting posttranscriptional regulation of cellulase gene expression as was suggested previously [61,62]. Consequently, PhLP1, GNB1 and GNG1 are important for regulation of cellulase gene expression by the heterotrimeric G-protein pathway. However, the function of PhLP1, GNB1 and GNG1 in regulation of cellulase gene expression is not dependent on light.

### PhLP1 is a regulator of pheromone expression

Targets of PhLP1-GNB1-GNG1 were found to include genes involved in sexual development, with the peptide pheromone precursor gene *hpp1 *[29] and the homologue of the yeast pheromone transporter gene *ste6 *(TR_62693 [GenBank: EGR48468.1]) as most interesting representatives. STE6 is involved in the secretion process of an a-type pheromone in *Schizophyllum commune *[63] and was shown to be required but not essential for mating in *Cryptococcus neoformans *[64]. The protein encoded by TR_62693 shows homology to *C. neoformans ste6 *with coverage of 91% and an E-value of 3e^-74 ^(blastX; NCBI Blast website [65]).

**Figure 9:**
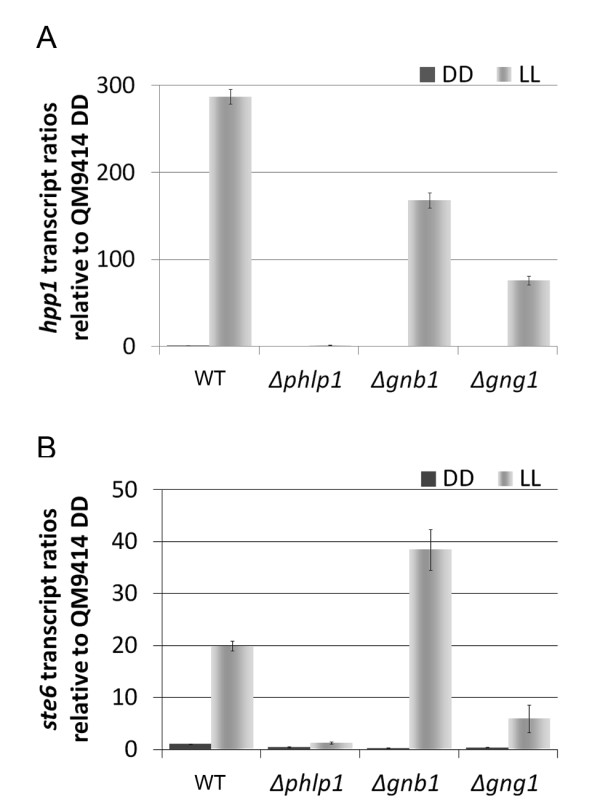
**Transcription of *hpp1 *and *ste6 *in Δ*phlp1*, Δ*gnb1 *and Δ*gng1***. Relative transcript abundance of the pheromone precursor gene *hpp1*, and the gene encoding its putative transporter *ste6*. Data were obtained by qRT-PCR and are given as relative values to the wild type strain QM9414 (WT) grown in darkness (DD).

Evaluation of microarray data by qRT-PCR confirmed considerably decreased transcript levels of *hpp1 *in Δ*phlp1 *and co-regulation of *hpp1 *with *ste6 *in Δ*phlp1 *and Δ*gng1 *(Figure 9). The data indicated that in darkness the pheromone precursor was downregulated to a basal level in all strains tested, confirming the importance of light for sexual reproduction [28]. Expression levels of *hpp1 *were significantly decreased in light in Δ*phlp1*, Δ*gnb1 *and Δ*gng1*, with lack of PhLP1 causing a decrease even to dark levels. Transcription of *ste6 *was enhanced upon deletion of *gnb1*, which might reflect a reaction to the decreased pheromone levels.

**Figure 10:**
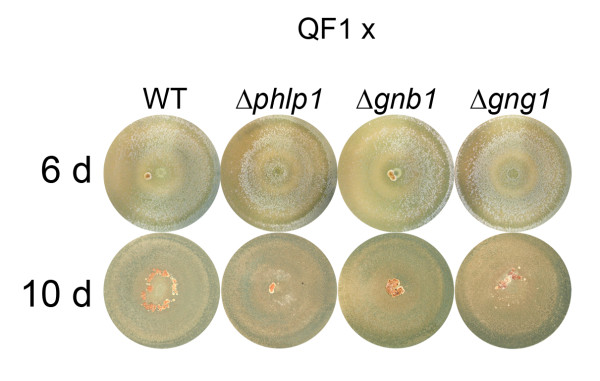
**Influence of PhLP1, GNB1 and GNG1 on sexual development**. Plates were inoculated with a mixture of equal amounts of spores from strain QF1 (MAT1-1), which serves as the sexually competent mating partner and the deletion strains Δ*phlp1*, Δ*gnb1 *and Δ*gng1 *along with the parental strain QM9414 (WT) as control (all MAT1-2). QF1 regained the ability for sexual development by crossing with the sexually competent wild type strain CBS999.97 and several rounds of backcrossing to restore the genetic background of QM9414 [88] Fruiting body formation is shown for 6 and 10 days after inoculation.

This influence of PhLP1, GNB1 and GNG1 on the pheromone precursor and transporter of the corresponding pheromone in submerged culture on cellulose prompted us to analyze sexual development under conditions appropriate for reproduction i. e. on solid medium (Figure 10). Despite considerably decreased transcript levels of *hpp1 *and *ste6 *in the Δ*phlp1 *strain in shake flasks on cellulose, this strain was still able to produce fruiting bodies. However, the amount of fruiting bodies formed by Δ*phlp1 *after 23 days was significantly lowered by 39% (p-value 0.045). Since the function of GNB1 and GNG1 is likely to be considerably affected by PhLP1, a role also for these factors in sexual development was expected. In Δ*gnb1 *(18.1% reduction, p-value 0.337) and Δ*gng1 *(1.8% reduction, p-value 0.922) no statistically significant decrease in fruiting body formation was observed. Additionally we found that in Δ*phlp1 *ascospore discharge was reduced by 85.9% (p-value 0.0049) and virtually abolished in Δ*gnb1 *(99.4%, p-value 0.00015). These results suggest a role for PhLP1 in development of female sexual structures and in reproductive efficiency.

## Discussion

The changing conditions between day and night or growth on the surface and in substrate require considerable physiological adjustments, the significance of which is so far poorly understood. Especially the differences in carbon source utilization and expression of the enzymes produced for this task in light and darkness are of utmost interest for both research and industry. In this study we investigated gene expression under conditions triggering production of plant cell wall degrading enzymes, the most important research focus of *T. reesei*. Constant conditions of illumination are used to reveal an influence of light especially on metabolic processes, while largely avoiding interference with circadian rhythmicity, which would be effective under the more natural conditions of 12:12 light-dark cycles. The analysis of short term light responsiveness of transcription of *phlp1, gnb1 *and *gng1 *(15 to 120 minutes of illumination) complemented long term light exposure experiments (72 hours of constant illumination or darkness).

**Figure 11:**
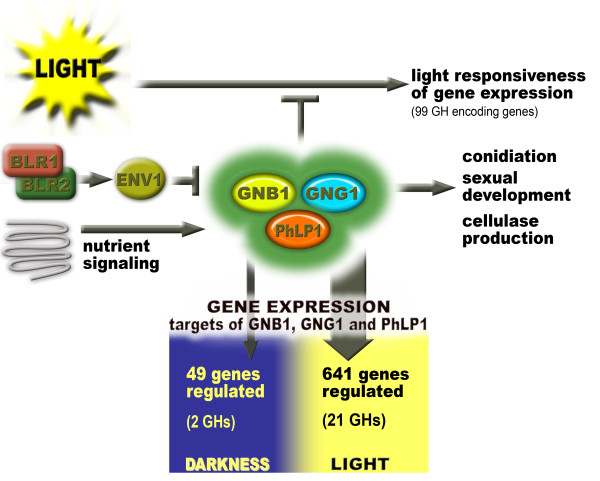
**Model for the function of PhLP1, GNB1 and GNG1**. GNB1, GNG1 and PhLP1 integrate signals received from the light response pathway and the heterotrimeric G-protein pathway, which predominantly transmits nutrient signals. The different transcript levels of genes between light and darkness in one strain (described as light responsiveness) of numerous genes, including 99 glycoside hydrolase (GH) encoding genes, is dampened by GNB1, GNG1 and PhLP1. Hence, GNB1, GNG1 and PhLP1 are assumed to transfer a positive light signal to the (presumably indirectly regulated) respective target genes, keeping their transcript levels elevated to the same extent as in darkness. A clear negative effect of ENV1 on *phlp1 *was observed, but this effect is not due to the regulation of ENV1 by BLR1 and BLR2, as they do not significantly alter the transcription of *gnb1, gng1 *or *phlp1*. Phenotypic effects of the deletion strains lacking *phlp1, gnb1 *and *gng1 *include conidiation and cellulase production, while sexual development was only influenced by GNB1 and PhLP1. The targets of GNB1, GNG1 and PhLP1 (i.e. genes differentially regulated in the deletion strains compared to the wild type) are predominantly found upon cultivation in light (13 genes upregulated, 628 genes downregulated) in contrast to a considerably smaller number in darkness (42 genes upregulated, 7 genes downregulated).

We elucidated the function of an important node between the light response pathway and nutrient signaling. Our results strongly point at a positive effect of the PhLP1-GNB1-GNG1 complex on a broad array of genes in light. While a strong impact of such a central signaling mechanism is not surprising, the huge amount of downregulated (or rather not upregulated) genes in light was unexpected. This finding supports a model in which distinct mechanisms are responsible for regulation of physiological processes in darkness and in light, with PhLP1-GNB1-GNG1 playing an important role for transmission of signals relevant to expression of hydrolytic enzymes and proteins involved in sexual reproduction in light (Figure 11). It may be even more astonishing that at least 23% of all genes can be regulated in a light dependent manner, as shown for strains lacking one of the signaling genes, an effect which was masked in the parental strain by tight regulation.

However, our results indicate that the pathway of heterotrimeric G-protein signaling represents an interactive regulatory network for signal transmission: Among the genes not upregulated in light in Δ*phlp1*, Δ*gnb1 *and Δ*gng1 *there were three G-protein coupled receptors and one regulator of G-protein signaling (RGS1). This finding could explain how the different relevance of signals in light and darkness as reported earlier for *T. reesei *[7,18,20,21] can result in altered regulation of output pathways. Signals integrated at the regulatory level of the G-protein beta and gamma subunits may adjust sensitivity of the organism to environmental signals by regulating the respective receptor. Transmission of a certain set of signals is modulated by an effect on RGS1 by action on the activity of different G-protein alpha subunits [66,67].

Interestingly, as seen for Δ*phlp1*, Δ*gnb1 *and Δ*gng1*, biomass formation in GNA1QL, a strain that expresses a constitutively activated G-protein alpha subunit (GNA1) is also reduced [20]. Identification of possible target genes of PhLP1-GNB1-GNG1 revealed *rgs1 *and a GPCR, containing an RGS domain, (TR_81383) as light independent targets. RGS proteins are regulators of G-proteins by activating the GTPase function and thereby control the G alpha activity negatively [17]. In *T. reesei *the number of genes comprising these functionalities is high (4 RGS-proteins and 3 GprK-type GPCRs; [36]) compared to one RGS-protein in *N. crassa *[68] or 4 RGS-proteins and one GprK-type GPCR in *A. nidulans *[69]. In accordance with our results, a study of the phosducin-like protein PhnA in *Aspergillus nidulans *showed a function of PhnA in regulation of the RGS protein FlbA, which controls the vegetative growth signaling pathway mediated by the G beta and gamma subunits [48]. Since deletion of *phlp1, gnb1 *or *gng1 *decreased the abundance of the *rgs1 *transcript and hence could lead to enhanced activity of GNA1, that is in agreement with our data and the hypothesis raised previously [20,48] that the growth defect observed upon alteration of GNA1 might at least in part be due to the function of the G-protein beta and gamma subunits.

An interesting question with respect to light dependent regulation of plant cell wall degrading enzymes (mainly glycoside hydrolases) is the correlation between growth rate of the fungus and biosynthesis of extracellular protein. The most elaborate studies on this topic in *T. reesei *were done under constant (chemostat) fermentation conditions: It was shown that upon growth under carbon limiting conditions with lactose as carbon source, slow growth correlates with high specific rates of extracellular protein synthesis [70]. However, the same study revealed that despite efficient synthesis of the major cellulase CEL7A/CBH1 at low growth rates, the protein secretion capacity limits protein production under these conditions. In terms of physiology, this phenomenon can be explained by the need to use hydrolytic enzymes to increase nutrient supply from insoluble extracellular substrates. Several studies showed an initial increase of extracellular protein production with increasing dilution rate and a decrease after reaching a peak was observed [70-73].

Studies using cellulose as carbon source and shake flasks for cultivation also do not provide a consistently direct correlation between growth rate and transcription of glycoside hydrolase genes: Growth and transcription of *cel7a/cbh1 *in the wild type strain QM9414 were in accordance with an indirect correlation between growth rate and cellulase transcription, as the lower growth rate of *T. reesei *in light is correlated with higher transcript levels of *cel7a/cbh1 *[34]. Since growth has been shown to represent an output pathway of light signaling [33] and the *T. reesei *photoreceptors BLR1 and BLR2 influence growth as well as cellulase gene expression [35], this connection is not surprising.

In the present genome wide study, we found both positive and negative light dependent regulation of an unexpectedly high number of glycoside hydrolases in QM9414 and mutant strains. Having in mind that the difference in growth observed in QM9414 between light and darkness was not similarly clear in the mutant strains, a strict correlation of glycoside hydrolase gene expression with growth rate cannot be proposed from the data of our study. Moreover, the fact that both positive and negative regulation of glycoside hydrolase genes in response to light was observed, refutes the hypothesis that the negative effect of light on the growth rate correlates with a consistently positive effect on transcription of glycoside hydrolase genes. Also, we found decreased, but still light-dependent regulation of transcript abundance of *cel7a/cbh1 *in Δ*phlp1*, Δ*gnb1 *and Δ*gng1 *(Figure 8A), although growth was less or not at all influenced by light in these strains. Consequently, we propose a model in which PhLP1, GNB1 and GNG1 represent crucial components of the pathway connecting light signaling to regulation of growth, which would explain the uncoupling of *cel7a/cbh1 *transcription from the growth rate. Alternatively, our data could indicate that the correlation of *cel7a/cbh1 *transcription with the growth rate is a specific event under the clearly defined conditions used in previous experiments, but not a general phenomenon. Likewise, an extrapolation of these findings to glycoside hydrolases in general would require a consistently indirect correlation of growth rate and transcription of glycoside hydrolase genes, which is clearly not the case. Therefore the assumption that transcription of glycoside hydrolase genes generally increases at lower growth rates is not supported. Our data rather point at a sophisticated adjustment of glycoside hydrolase gene expression by up- and downregulation of certain groups which is triggered by PhLP1, GNB1 and GNG1. While we found growth to be regulated in response to light in any case, we could not obtain conclusive evidence that this might be achieved exclusively by regulation of substrate degradation efficiency (glycoside hydrolase biosynthesis) in *T. reesei*, but must (also) be governed by other pathways.

The positive influence of PhLP1 on *ste6 *and *hpp1 *transcription added another piece of the puzzle to the regulatory mechanisms in G-protein signaling. The decreased fruiting body formation (by 39%) and ascospore discharge (by 86%) in the *phlp1 *deletion strain corresponds with the lower transcript levels of *hpp1 *and *ste6 *in this strain, as it was shown that *hpp1 *is necessary for male fertility in *T. reesei *QM9414 [29]. This result reflects a crucial role of PhLP1 in sexual development, which is in accordance with the function of its orthologue in *A. nidulans *[48]. Nevertheless, the mating defect of the strain lacking GNB1 was even more severe, but with virtually abolished ascospore discharge concerns a different aspect of mating. Although our study was focused on elucidation of the interrelationship between light response and nutrient signaling, the fact that sexual development of *T. reesei *is dependent on light [28] draws a logical connection of these signaling pathways to sexual development. Accordingly, in *Schizosaccharomyces pombe *the nutrient regulated G-protein cAMP pathway and the pheromone regulated MAP kinase signaling pathway are interconnected and hence mating is also controlled by the availability of nutrients in this fungus (reviewed in [16]). An involvement of the G-protein beta subunit in sexual development is also known from other fungi. In *N. crassa *it was shown that the deletion of the gene encoding the G-protein beta subunit GNB-1 results in a female sterile, but male fertile strain with small perithecia and no ascospore ejection [74]. In *Aspergillus nidulans *lack of the G-beta subunit caused an increase in Hülle cell formation, which supports fruiting body formation, but no cleistothecia could be found [75] and in the basidiomycete *Cryptococcus neoformans *G-protein beta is required for haploid fruiting and fertility [76].

## Conclusions

In summary, with PhLP1-GNB1-GNG1 we could identify a further step in the signaling cascade aimed at light-modulated cellulase gene expression (Figure 11). Thereby the targets among substrate degrading enzymes (glycoside hydrolases) are more widespread than expected. The considerable number of glycoside hydrolases showing differential transcription between light and darkness, which is in many cases subject to regulation by PhLP1-GNB1-GNG1, reflects the high significance of light for fungi also in terms of substrate degradation. Interestingly, also G-protein coupled receptors and regulators of G-protein function (RGS-proteins) are among the targets of PhLP1-GNB1-GNG1 - which can be considered to act somewhat at the downstream end of the G-protein pathway. Hence we conclude that the pathway of heterotrimeric G-protein signaling does not strictly act as a cascade but rather represents an interactive network for signal integration and transmission. A function of PhLP1 and GNB1 also in mating efficiency confirms PhLP1 and its immediate targets as crucial nodes in adjusting the physiology of *T. reesei *to the central determinants of life in nature: the rotation of earth, acquisition of nutrients and reproduction.

## Methods

### Strains, plasmids and culture conditions

In this study *Trichoderma reesei *QM9414 (ATCC 26921) was used as the parental strain. All strains were kept on 3% (w/v) malt extract agar unless otherwise noted. For selection of positive transformants the medium was supplemented with hygromycin B (50 μg/ml) (Roth, Karlsruhe, Germany). For quantitative RT-PCR analysis, the strains were grown in liquid culture in 200 ml Mandels-Andreotti minimal medium [77] supplemented with 0.1% (w/v) peptone to induce germination and with 1% (w/v) carbon source at 28°C on a rotary shaker (200 rpm). The cultures were kept either in constant darkness (DD) or constant light (LL, 25 μmol photons m^-2 ^s^-1^; 1800 lux) and microcrystalline cellulose (# 1402; SERVA, Heidelberg, Germany) was used as carbon source. For evaluation of light response, strains were pregrown in darkness for 24 hours (24DD) and exposed to light (1800 lux) for different time periods (indicated as minutes DL) upon growth on 1% (w/v) glycerol (Merck, Darmstadt, Germany) as carbon source. Harvesting of the mycelia in dark conditions was performed using safe-red-light (darkroom lamp, Philips PF712E, red, E27, 15 W). For plate assays, strains were grown on solid malt extract agar for 3 days in constant light (300 lux) or constant darkness.

*Escherichia coli *JM109 was used for the propagation of vector molecules and DNA manipulations [78].

### Construction of *T. reesei *Δ*phlp1*, Δ*gnb1 *and Δ*gng1 *strain

Sequences of oligonucleotides used in this study are given in table 1. The protoplast method was used for *T. reesei *transformation [79,80] of the vector constructs described below. QM9414 was used as the parental strain in all cases. Putatively positive transformants were purified by at least two rounds of single spore isolation and recultivation on selective medium prior to analysis for successful deletion of the target gene. pBSXH, which comprises the *hph*-marker cassette [34], served as backbone for construction of the deletion vectors described in the following.

**Table 1:** Sequences of oligonucleotides used in this study

Purpose	Oligonucleotide	Sequence
*phlp1*	PHD2DEL5F	5' ATGCGGCCGCTGCTCGTAAAGAGGTGCAG 3'
	
5' region	PHD2DEL5R	5' ATACTAGTGCTGAGCGCTGTTATATAGATG 3'

*phlp1*	PHD2DEL3F	5' ATCTCGAGCAATGATTTGCACAACTC 3'
	
3' region	PHD2DEL3R	5' ATGGTACCGCTAGCAGCATCAACACCTCTCTAC 3'

*gnb1*	DELgnb15F	5' ATACTAGTCCAGGTATCAAGGCTCTCATC 3'
	
5' region	DELgnb15R	5' ATGATATCGGGAGAAGACGATGGAGATG 3'

*gnb1*	DELgnb13F	5' ATCTCGAGTCATCACCATCCGCATTC 3'
	
3' region	DELgnb13R	5' ATGGTACCCAGACTTTGACATGCCAATG 3'

*gng1*	gng1DEL5F	5' ATACTAGTCCACTGCTAATTATCCGC 3'
	
5' region	gng1DEL5R	5' ATCCCGGGCGGAGGGTGAATTAAAGG 3'

*gng1*	gng1DEL3F	5' ATCTCGAGATCGATGATGAAGGAGCTACTG 3'
	
3' region	gng1DEL3R	5' ATGGTACCTGGCATGAGCTTTCAACTTC 3'

*phlp1*	PHD2cDNAF	5' TATATAACAGCGCTCAGC 3'
	
cDNA	PHD2cDNAR	5' AGAATGCTTTAAGAGTTGTG 3'

*gnb1*	gnb1cDNA1F	5' ATACTAGTTCGCCCGCCTCCCATCTC 3'
	
cDNA	gnb1cDNA1R	5' ATAGGCCTAGATATGCGTAGTCGGGTGTCC 3'

*gng1*	gng1cDNA1F	5' ATGTCGACGCATCAGTTCCAACTCGAC 3'
	
cDNA	gng1cDNA1R	5' ATAGGCCTAAATCAAAGCGATCCCAC 3'

*cbh1*	RTcbh1F	5' ACCGTTGTCACCCAGTTCG 3'
	
qRT-PCR	RTcbh1R	5' ATCGTTGAGCTCGTTGCCAG 3'

*phlp1*	RTphd2F	5' GACAGGAGCTCGAGAAGGAAG 3'
	
qRT-PCR	RTphd2R	5' CAAAGACGGCAACGGTAGTG 3'

*gnb1*	RTgnb1F	5' CATCAACGACCGAAGCATC 3'
	
qRT-PCR	RTgnb1R	5' GCAGGCACCAGAAATGAAG 3'

*gng1*	RTgng1F	5' CGTACTGCAATGGCACAAGAG 3'
	
qRT-PCR	RTgng1R	5' GGATTGCTGAGGCGCATAG 3'

rpl6e	RTL6eF1	5' GATACGTCATCGCCACCTCC 3'
	
qRT-PCR	RTL6eR1	5' CTTCTCCTTGGCCTTCTCG 3'

*cbh2 *Terminator	cbh2T_F1	5' GGCTTGCTCGCTGACTGATAC 3'
	
qRT-PCR	cbh2T_R1	5' GAGGGAGACGAGGTTGTGATG 3'

*hph*	HPH_F1	5' GATGTAGGAGGGCGTGGATATG 3'
	
qRT-PCR	HPH_R1	5' GGGAGATGCAATAGGTCAGG 3'

For amplification of the 5' flanking region of *phlp1 *by PCR, primers PHD2DEL5F and PHD2DEL5R were used. The 3' region was amplified using primers PHD2DEL3F and PHD2DEL3R. First, the 3' fragment (883 bp) was integrated into pBSXH digested with *Xho*I and *Acc*65I and purified (all restriction enzymes by Fermentas, Vilnius, Lithuania) resulting in pDphlp3. Then, the 971 bp 5' fragment was cloned into pDphlp3 using *Not*I and *Spe*I to obtain pDELphlp1. A linear fragment of the deletion cassette for transformation was obtained by excision of the deletion cassette using *Not*I and *Acc*65I and transformed into QM9414.

Putative transformants were checked for deletion by PCR, using the primers PHD2cDNAF/PHD2cDNAR, which are located adjacent to the deleted open reading frame. Thereby a 2900 bp fragment indicated successful deletion of *phlp1 *in the respective transformant while the presence of the wild type fragment (990 bp) revealed ectopic or no integration of the deletion cassette (data not shown).

The 5' flanking region of *gnb1 *was amplified by PCR using the primers DELgnb15F and DELgnb15R. For amplification of the 3' flanking fragment we used DELgnb13F and DELgnb13R. The 1092 bp 3' fragment was inserted first into the vector pBSXH with *Xho*I and *Acc65*I to obtain pDgnb13. Then the 1010 bp fragment of the 5' flanking region was integrated into pDgnb13 using *Spe*I and *EcoR*V to obtain pDELgnb1. The vector was linearized with *Spe*I for transformation.

Primers gnb1cDNA1F and gnb1cDNA1R were used for diagnostic PCR. Positive transformants showed a band with 2898 bp length, in contrast to the wild type fragment of 1679 bp (data not shown).

For deletion of *gng1 *the 3' and the 5' flanking sequences were amplified with primers gng1DEL5F and gng1DEL5R for the 5' flanking sequence. For the 3' region gng1DEL3F and gng1DEL3R were used. The 1023 bp fragment of the 5' flanking region was ligated into pBSXH with *Spe*I and *Sma*I resulting in pDgng15. Then, the 1037 bp fragment of the 3' flanking region was inserted into pDgng15 with *Xho*I and *Acc65*I to obtain pDELgng1. *Spe*I and *Acc*65I were used for excision of the deletion cassette to be used for transformation.

The Δ*gng1 *transformant was confirmed by using primers gng1cDNA1F/gng1cDNA1R. We obtained a fragment of 2924 bp indicating successful deletion. The corresponding fragment of the parental strain (471 bp) was absent (data not shown).

### Retransformation of knockout mutants

For complementation of the deletion mutants the vector pBSamdS [29], which comprises the *amd*S marker cassette and the pBluescript SK+ backbone, was used. The complementation fragment for Δ*phlp1 *was amplified with primers PHD2DEL5F and PHD2DEL3R. The fragment was ligated into pBSamdS using *Acc*65I and *Sal*I. The vector was linearized with *Kpn*I for transformation.

The fragments for the *gnb1 *and *gng1 *retransformation vectors were amplified using the primer pairs DELgnb15F/DELgnb13R or gng1DEL5F/gng1DEL3R, respectively, and ligated into pGEM-T Easy (Promega, Madison, USA). The 3732 bp fragment comprising the *gnb1 *gene and the 2445 bp fragment for *gng1 *were then excised from pGEM-T Easy and integrated into pBSamdS using *Not*I restriction sites. For linearization, *Apa*I was used in both cases.

All retransformed strains showed a rescued phenotype (Additional file 3, Figure S4), which clearly differs from the deletion strains (Figure 3).

### Nucleic acid isolation and determination of copy number

Fungal mycelia were harvested by filtration, washed with tap water and frozen in liquid nitrogen. Extraction of genomic DNA and total RNA was performed by protocols described previously [21,81]. DNA and RNA concentrations were measured using the Nanodrop ND-1000 spectrophotometer (PEQLAB, Erlangen, Germany).

For determination of copy numbers of the deletion cassette in Δ*phlp1*, Δ*gnb1 *and Δ*gng1 *genomic DNA samples were prepared for these mutants, the parental strain QM9414 as negative control and from the strain GNA3QLE, for which a single integration of the *hph *deletion cassette at the locus was shown by Southern blotting [21] as positive control. The Bio-Rad SYBR Green Mix and the IQ5 ICycler system (Bio-Rad, Hercules, USA) were used for quantitative PCR. gDNA was diluted to a concentration of 2 and 0.2 ng/μl for each DNA sample. Using qPCR and primers HPH_F1/HPH_R1, the number of *hph *copies relative to the reference gene *l6e *was determined according to the Pfaffl-method [82]. To confirm the results obtained with *hph *primers, we also quantified the *cel6a/cbh2 *terminator sequence, which is located in the *hph*-marker cassette [80] using primers cbh2T_F1 and cbh2T_R1. Consequently, the parental strain has one copy of the *cbh2 *terminator and with each integrated deletion cassette the number increases by one (Additional file 3, Figure S5).

As expected we detected one copy of the *cel6a/cbh2 *terminator and no *hph *cassette in QM9414 as well as two copies of the *cel6a/cbh2 *terminator and one copy of the *hph *cassette in GNA3QLE. Δ*phlp1 *and Δ*gnb1 *were found to comprise a single copy of the *hph *cassette, while Δ*gng1 *contained three copies (Additional file 3, Figure S5). However, a different deletion mutant of *gng1 *showed the same phenotype and in both cases wild type behavior (biomass formation, conidiation and sexual development (Additional file 3, Figures S1B, S3B, S4 and S6, respectively) was rescued by retransformation. Hence the additional copies in Δ*gng1 *did not influence our analysis.

### Phenotypic analyses

For biomass analysis strains were kept on malt extract agar plates in darkness prior to inoculation of Mandels-Andreotti minimal medium with microcrystalline cellulose or glycerol as carbon source. For analysis of biomass obtained from fungi grown on cellulose, mycelia were harvested after 72 hours of incubation and analyzed as described earlier [34]. For analysis of the biomass in the presence of glycerol, cultures were kept in constant darkness or constant light, respectively, for 20 h, 25 h and 30 h. The mycelium was harvested using pre-weighed glass microfiber filters (Cat. No. 1822-055, Whatman, Kent, UK), washed with tap water, dried at 80°C for two days and analyzed.

For measurement of conidiation, malt extract agar plates were inoculated and incubated in constant light or constant darkness for 3 days. Spores were harvested with 1 ml of sterile tap water and filtered with glass wool. The concentration was determined by measurement of the optical density with a spectrophotometer (Helios, Thermo Fisher Scientific, Waltheim, USA) at a wavelength of 600 nm and analyzed using a standard curve. The results are given as the number of spores per cm^2 ^covered with mycelia.

### Analysis of enzyme activity

Strains were grown for 72 hours in constant light or darkness on cellulose as carbon source as described above. Specific filterpaper activity (FPA) was measured as described previously [61]. Biomass formation for calculation of specific FPA was determined as described in [34]. At least three technical and two biological replicates were used to ensure statistical significance.

### cDNA preparation for quantitative reverse transcription PCR and microarray analysis

Total RNA was isolated and treated as described by [21]. cDNA synthesis was performed using the RevertAid-H^- ^First Strand cDNA Synthesis Kit (Fermentas) and Oligo-d(T)-Primers for quantitative RT-PCR analysis and Random Hexamer Primers for microarray experiments following the manufacturer's instructions. The Bio-Rad SYBR Green Mix and the IQ5 ICycler system (Bio-rad) were used for qRT-PCR as described previously [21]. All experiments were done in technical triplicate with at least two different biological replicates. The primers used for qRT-PCR are given in table 1 along with the corresponding product length. We used transcription of the ribosomal gene *rpl6e *for normalization of the qRT-PCR data [21,83,84]. This gene was shown to be a suitable reference gene for light/darkness transcription analysis in *T. reesei *[21].

### Microarray experiment and data analysis

Microarray experiments were performed using the gene expression full service provided by Roche-NimleGen (Madison, USA) with two biological replicates. Oligonucleotide arrays were designed as custom arrays by Roche-NimbleGen based on the *Trichoderma reesei *v2.0 genome sequence using the 4 × 72000 format, which allowed for an average of 7 probes (60 mer) per gene model. The Expression Full Service provided by Roche-NimbleGen included standardized single channel hybridization of slides, which enables batch to batch comparison and determination of gene specific expression values. Raw data have been deposited at Gene Expression Omnibus under accession number GSE27581. Analysis of microarray data and GSEA (Gene set enrichment analysis) were performed using the Partek Genomics Suite 6.5 (Partek Inc., St. Louis, USA), applying ANOVA (analysis of variation) for identification of statistically significant differentially expressed genes. ANOVA generalizes the t-test to more than two groups and consequently allows any number of categorical effects. Therefore the chance to commit a type I error ("a positive assumption is false") by doing multiple two-sample t-tests is avoided. The combined p-value for significant regulation due to different light conditions and strains was set to < 0.1. All comparisons of transcript abundance refer to an at least two-fold significantly differential transcription unless noted otherwise. For cluster analysis the open source software HCE 3.5 [85,86] was used.

For evaluation of results the community annotation including GO (Gene Ontology) classifications, as available at the *T. reesei *genome database v2.0 [87], was used. Additionally, annotations of all glycoside hydrolases and of genes involved in signal transduction, light response or sexual development were revised and amended manually if necessary.

### Analysis of sexual development

Conidiospores (1*10^6^) of Δ*phlp1*, Δ*gnb1*, Δ*gng1*, or the parental strain QM9414 were mixed with 1*10^6 ^conidiospores of QF1, a strain derived from QM9414, which has regained the ability for sexual development by backcrossing [88] and used for inoculation of a malt extract agar plate (3%). Strains were incubated for 23 days in daylight at room temperature until the ejection of ascospores was finished. Strains were phenotypically analyzed 6 and 10 days after inoculation in order to evaluate efficiency of fruiting body formation. After ascospore discharge on day 23, all fruiting bodies were harvested from the plates, dried at 70°C for 24 hours and the dry weight was determined. Five replicates were analyzed for each strain. The data was evaluated by ANOVA and the significant p-value was set to p < 0.05 for differences between the parental strain QM9414 and the deletion strains.

The ascospores were harvested from the lid of the plates with 1 ml of sterile tap water and their concentration was determined by measurement of the optical density with a spectrophotometer. ANOVA with a p-value p < 0.05 was used to analyze the data. For confirmation of ascospore measurement, 10 μl of the suspension was inoculated on malt extract agar plates and the number of colonies formed was counted (data not shown).

## Authors' contributions

DT performed the experiments, interpreted the results and drafted the manuscript. CPK participated interpretation of results. MS conceived of the study, interpreted the results and wrote the final version of the manuscript. All authors read and approved the final manuscript.

## Supplementary Material

Additional file 1 **Light responsiveness of gene transcription as influenced by PhLP1, GNB1 or GNG1**. Genes at least two-fold up- or downregulated in light compared to darkness in QM9414 and deletion strains Δ*phlp1*, Δ*gnb1 *and Δ*gng1*. Click here for file

Additional file 2 **Enrichment analysis of genes regulated in response to light**. (A) Functional enrichment of genes two-fold upregulated in light compared to darkness. (B) Functional enrichment of genes two-fold downregulated in light compared to darkness. For Gene set enrichment analysis (GSEA), the threshold for significant enrichment was set to a p-value of lower than 0.005. Higher Enrichment Scores (ES) reflect more significant enrichment of the respective function. Click here for file

Additional file 3 **Figure S1 - Analysis of sporulation**. Figure S2 - Analysis of hyphal extension rates. Figure S3 - Biomass formation on glycerol. Figure S4 - Phenotypes of complemented knockout strains. Figure S5 - Determination of copy numbers of deletion cassettes in deletion mutants. Figure S6 - Crossings of complemented knockout strains with QF1. Click here for file

Additional file 4 **Regulation of glycoside hydrolase genes in QM9414 and the deletion strains Δ*phlp1*, Δ*gnb1 *and Δ*gng1***. Click here for file

Additional file 5 **Targets of PhLP1**. Genes at least two-fold differentially regulated in the Δ*phlp1 *deletion strain compared to QM9414 in light and darkness. Click here for file

Additional file 6 **Targets of GNB1**. Genes that are at least two-fold differentially regulated in the Δ*gnb1 *deletion strain compared to QM9414 in light and darkness. Click here for file

Additional file 7 **Targets of GNG1**. Genes at least two-fold differentially regulated in the Δ*gng1 *deletion strain compared to QM9414 in light and darkness. Click here for file

Additional file 8 **Overlapping Targets of PhLP1, GNB1 and GNG1**. Genes at least two-fold differentially regulated in Δ*phlp1*, Δ*gnb1 *and Δ*gng1 *compared to QM9414 in light and darkness. Click here for file
