# Supplementary material for: The phosducin-like protein PhLP1 impacts regulation of glycoside hydrolases and light response in Trichoderma reesei
Source: BMC Genomics. 2011 Dec 19;12:613. doi: 10.1186/1471-2164-12-613 (PMC3267782; doi:10.1186/1471-2164-12-613)
Supplement: Additional file 2 — Enrichment analysis of genes regulated in response to light. (A) Functional enrichment of genes two-fold upregulated in light compared to darkness. (B) Functional enrichment of genes two-fold downregulated in light compared to darkness. For Gene set enrichment analysis (GSEA), the threshold for significant enrichment was set to a p-value of lower than 0.005. Higher Enrichment Scores (ES) reflect more significant enrichment of the respective function. [file 1471-2164-12-613-S2.PDF]

**Additional file 2, Table S1A**

GO Enrichment analysis of genes 2fold upregulated in light compared to darkness in the wild type QM9414

| Functional category | Enrichment Score |
|---|---|
| **BIOLOGICAL PROCESS** | |
| ► **Carbon utilization** | **8,85** |
| ► Metabolic process | 0,81 |
| ►► Catabolic process | |
| ►►► carbohydrate catabolic process | |
| ►►►► **polysaccharide catabolic process** | **44,24** |
| ►► primary metabolic process | |
| ►►► **carbohydrate metabolic process** | **25,03** |
| ► biological regulation | |
| ►► regulation of molecular function | |
| ►►► regulation of catalytic activity | |
| ►►►► **regulation of oxidoreductase activity** | **25,41** |
| ► Establishment of localization | |
| ►► transport | 1,4 |
| ►►► carbohydrate transport | 1,68 |
| ►►►► **phosphoenolpyrouvate-dependent sugar phosphotransferase system** | **5,92** |
| ►►► ion transport | |
| ►►►► anion transport | |
| ►►►►► inorganic anion tranport | |
| ►►►►►► **sulfate transport** | **11,72** |
| **MOLECULAR FUNCTION** | |
| ► transporter activity | 1,32 |
| ►► transmembrane transporter activity | |
| ►►► active transmembrane transporter activity | |
| ►►►► secondary active transmembrane transporter activity | |
| ►►►►► **secondary active sulfate transmembrane transporter activity** | **11,72** |
| ► sequence-specific DNA binding transcription factor activity | 0,52 |
| ► catalytic activity | 0,28 |
| ►► hydrolase activity | 0,56 |
| ►►► hydrolase activity, acting on glycosyl bonds | |
| ►►►► **hydrolase activity, hydrolyzing O-glycosyl compounds** | **32,85** |
| ►►►►► **cellulase activity** | **11,72** |
| ►► lyase activity | |
| ►►► carbon-carbon lyase activity | |
| ►►►► **DNA photolyase activity** | **11,72** |
| ►►►►► **deoxyribodipyrimidine photo-lyase activity** | **11,72** |
| ►► oxidoreductase activity | 3,09 |
| ►►► steroid dehydrogenase activity | 0,76 |
| ►►► monooxygenase activity | 0,19 |
| ► binding | |
| ►► nucleic acid binding | 1,65 |
| ►►► DNA binding | 0,29 |
| ►► nucleotide binding | 0,61 |
| ►► protein binding | 0,12 |
| ► carbohydrate binding | |
| ►► **sugar binding** | **8,85** |
| ►► polysaccharide binding | |
| ►►► **cellulose binding** | **36** |
| **CELLULAR component** | |
| ► **extracellular region** | **20,18** |

**Additional file 2, Table S1B**

GO Enrichment analysis of genes 2fold upregulated in light compared to darkness in the wild type QM9414

| Functional category | Enrichment Score |
|---|---|
| **BIOLOGICAL PROCESS** | |
| ► metabolic process | 0,71 |
| ►► biosynthetic process | 0,51 |
| ►►► **lipid biosynthetic process** | **14,66** |
| ►► small molecule metabolic process | |
| ►►► cellular ketone metabolic process | |
| ►►►► oxoacid metbalic process | |
| ►►►►► carboxylic acid metabolic process | |
| ►►►►►► carboxylic acid biosynthetic process | |
| ►►►►►►► **fatty acid biosynthetic process** | **14,54** |
| ►► nitrogen compound metabolic process | 2,09 |
| ► establishment of localization | |
| ►► **transport** | **9,19** |
| ►►► **nucleobase, nucleoside, nucleotide and nucleic acid transport** | **11,08** |
| ►►► carbohydrate transport | 2,41 |
| ►►► peptide transport | |
| ►►►► **oligopeptide transport** | **14,66** |
| **MOLECULAR FUNCTION** | |
| ► **transporter activity** | **9,99** |
| ►► substrate-specific transporter activity | |
| ►►► **acyl carrier activity** | **14,66** |
| ► structural molecule activiy | 3,39 |
| ►► structural constituent of ribosome | 0,52 |
| ► catalytic activity | 1,1 |
| ►► **transferase activity** | **8,38** |
| ►► oxidoreductase activity | 1,31 |
| ►►► steroid dehydrogenase activity | 1,12 |
| ►►► monooxygenase activity | 0,55 |
| ►►► oxidoreductase activity, acting on paired donors, with incorporation or reduction of molecular oxygen | |
| ►►►► oxidoreductase activity, acting on paired donors, with incorporation of reduction of molecular oxygen, NADH or NADPH as one donor, and incorporation of one atom of oxygen | |
| ►►►►► **salicylate 1-monooxygenase activity** | **20,77** |
| ►►► oxidoreductase activity, acting on the CH-NH2 group of donors | |
| ►►►► oxidoreductase activity, acting on the CH-NH2 group of donors, oxygen as acceptor | |
| ►►►►► **D-amino-acid oxidase activity** | **14,66** |
| ►►►►► **aspartate oxidase activity** | **14,66** |
| ►►►►►► **D-aspartate oxidase activity** | **14,66** |
| ►► hydrolase activity | 0,29 |
| ►►► **hydrolase activity, acting on ester bonds** | **14,98** |
| ►►►► phosphoric ester hydrolase activity | 4,46 |
| ►►►► nuclease activity | 2,36 |
| ►►►►► exonuclease activity | 4,46 |
| ►►► hydrolase activity, acting on carbon-nitrogen (but not peptide) bonds | 2,7 |
| ► binding | 0,89 |
| ►► **cofactor binding** | **12,77** |
| ►► nucleic acid binding | 1,06 |
| ►►► DNA binding | 0,23 |
| ►► nucleotide binding | 0,48 |
| ► sequence-specific DNA binding transcription factor activity | 0,72 |
