# Supplementary material for: The phosducin-like protein PhLP1 impacts regulation of glycoside hydrolases and light response in Trichoderma reesei
Source: BMC Genomics. 2011 Dec 19;12:613. doi: 10.1186/1471-2164-12-613 (PMC3267782; doi:10.1186/1471-2164-12-613)
Supplement: Additional file 3 — Figure S1 - Analysis of sporulation. Figure S2 - Analysis of hyphal extension rates. Figure S3 - Biomass formation on glycerol. Figure S4 - Phenotypes of complemented knockout strains. Figure S5 - Determination of copy numbers of deletion cassettes in deletion mutants. Figure S6 - Crossings of complemented knockout strains with QF1. [file 1471-2164-12-613-S3.PDF]

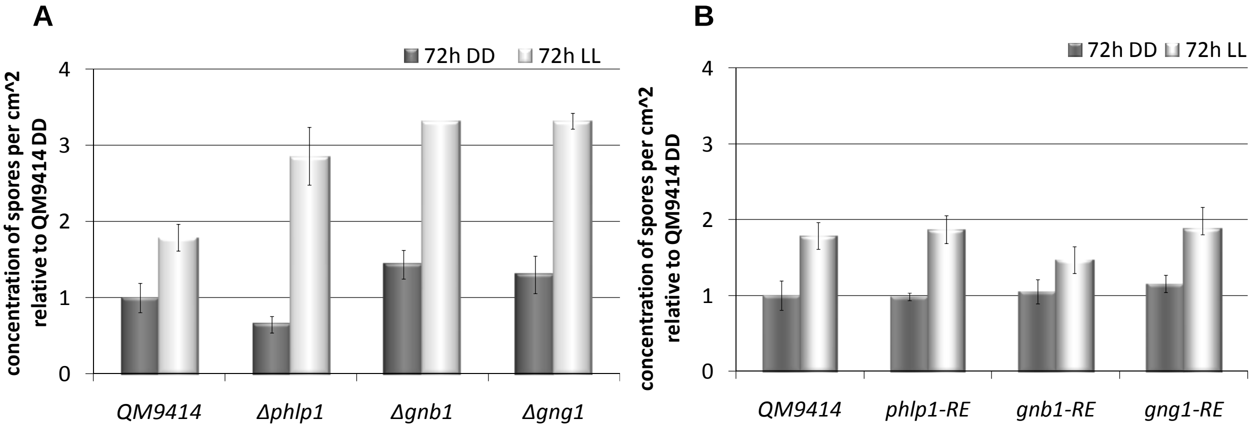

Figure S1 – **Analysis of sporulation**

Strains were cultivated at 28°C in constant light or darkness for 3 days. A: Parental strain QM9414 and deletion mutants Δ*phlp1*, Δ*gnb1* and Δ*gng1*. B: Parental strain QM9414 and the respective complemented knock-out mutants *phlp1*-RE, *gnb1*-RE and *gng1*-RE, which regained behaviour of the parental strain by retransformation of the original gene.

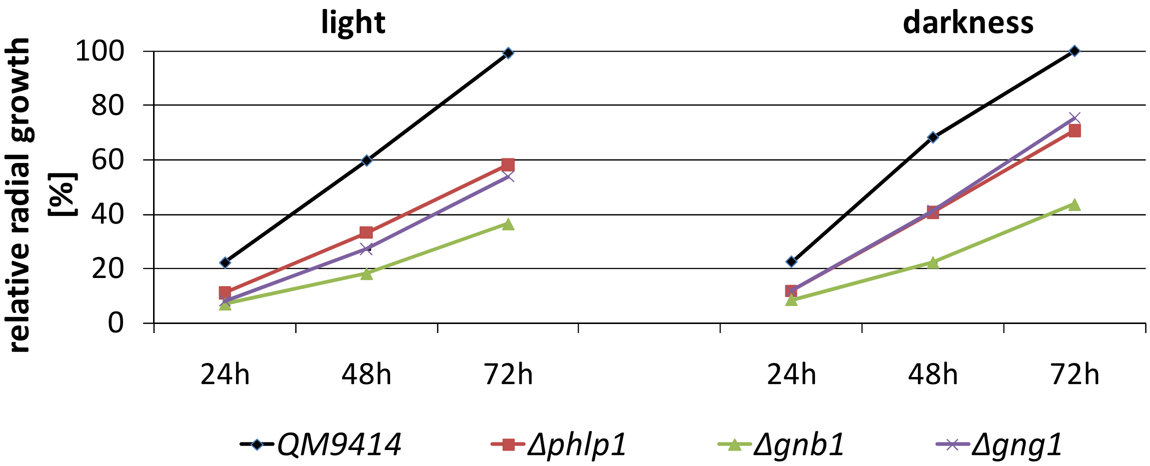

Figure S2 – **Analysis of hyphal extension rates**

Comparison of hyphal extension rates of the deletion strains Δ*phlp1*, Δ*gnb1* and Δ*gng1* with the parental strain QM9414 on malt extract agar plates (3 % w/v) in constant light or darkness.

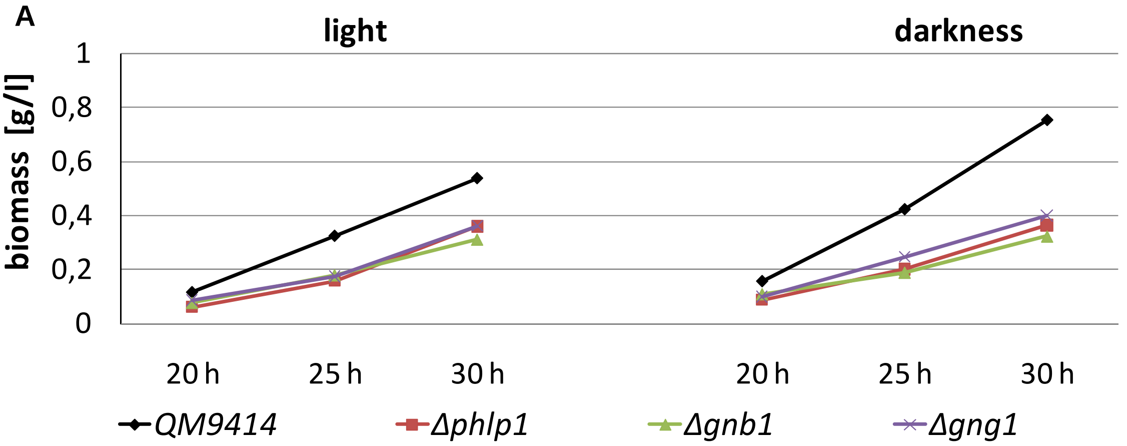

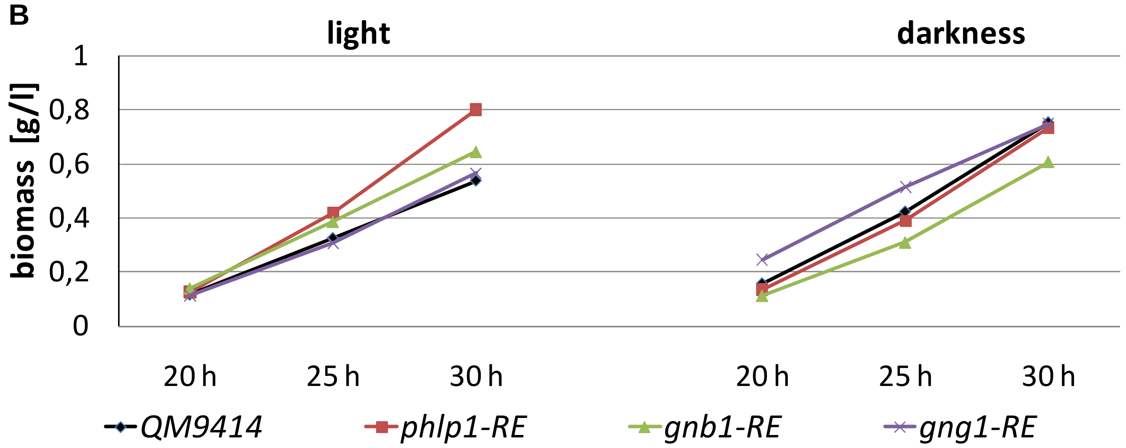

Figure S3 – Biomass formation on glycerol

Strains were grown on Mandels-Andreotti minimal media with 1% (w/v) glycerol as carbon source in constant light or constant darkness and biomass was determined after 20 hours, 25 hours and 30 hours. A: Comparison of the biomass formation of the deletion strains Δ*phlp1*, Δ*gnb1* and Δ*gng1* with the parental strain QM9414 in liquid media. B: Comparison of the respective complemented mutant strains *phlp1*-RE, *gnb1*-RE and *gng1*-RE with the parental strain QM4914.

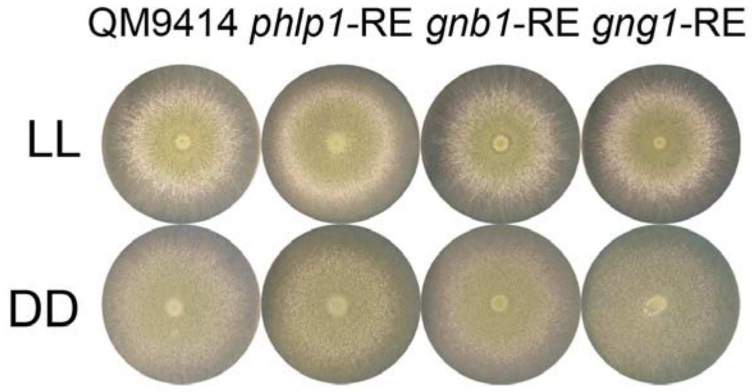

Figure S4 – Phenotypes of complemented knockout strains

Strains were kept at 28°C in constant light or darkness on malt extract agar plates for 3 days.

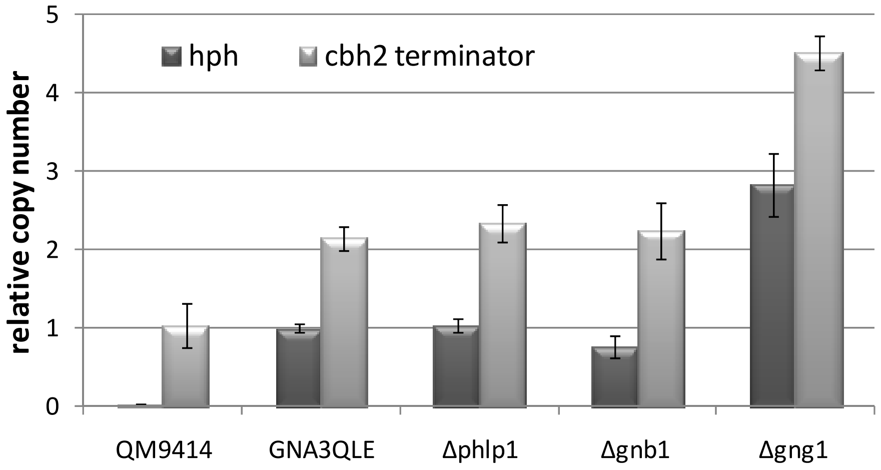

Figure S5 - Determination of copy numbers of deletion cassettes in deletion mutants

Copy number was determined by quantitative PCR. *L6e* was taken as reference gene and the amount of integrated *hph* is relative to GNA3QLE (positive control, one deletion cassette integrated). The amount of integrated *cbh2* terminator is given relative to the parental strain QM9414.

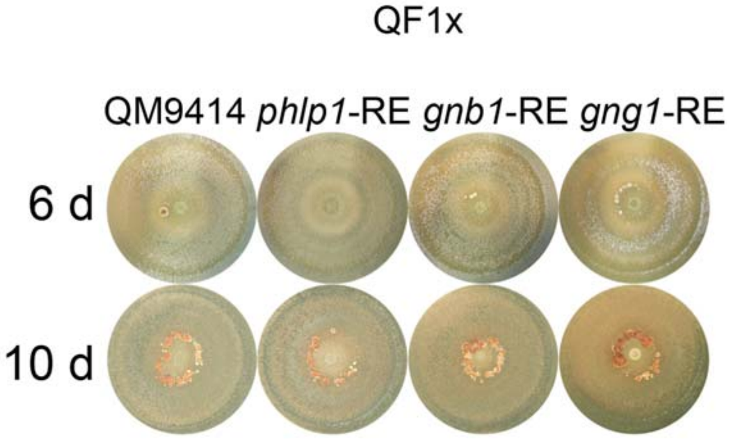

Figure S6 – Crossings of complemented knockout strains with QF1

Equal amounts of conidospores of QM9414, *phlp1*-RE, *gnb1*-RE or *gng1*-RE (all MAT1-2) and QF1 (MAT1-1, sexually compentent strain derived from QM9414) were combined and inoculated on malt extract agar plates. Sexual development was monitored for 23 days and fruiting body formation is shown for 6 and 10 days after inoculation.
